# Structure, activity and function of the lysine methyltransferase SETD5

**DOI:** 10.3389/fendo.2023.1089527

**Published:** 2023-02-17

**Authors:** Mingyang Li, Yanan Hou, Ziwei Zhang, Bowen Zhang, Ting Huang, Aiqin Sun, Genbao Shao, Qiong Lin

**Affiliations:** Department of Basic Medicine, School of Medicine, Jiangsu University, Zhenjiang, China

**Keywords:** SETD5, lysine methyltransferase, posttranslational modifications, methylation, neurodevelopmental disorder (NDD), cancer

## Abstract

SET domain-containing 5 (SETD5) is an uncharacterized member of the protein lysine methyltransferase family and is best known for its transcription machinery by methylating histone H3 on lysine 36 (H3K36). These well-characterized functions of SETD5 are transcription regulation, euchromatin formation, and RNA elongation and splicing. SETD5 is frequently mutated and hyperactive in both human neurodevelopmental disorders and cancer, and could be down-regulated by degradation through the ubiquitin-proteasome pathway, but the biochemical mechanisms underlying such dysregulation are rarely understood. Herein, we provide an update on the particularities of SETD5 enzymatic activity and substrate specificity concerning its biological importance, as well as its molecular and cellular impact on normal physiology and disease, with potential therapeutic options.

## 1 Introduction

Methyltransferases are a superfamily of enzymes very present in nature, acting in the methylation of proteins, nucleic acids, and small molecules (1, 2). These enzymes work by catalyzing a methyl group for a receptor molecule, generating S-adenosylmethionine (SAM) and a modified methylated molecule (3). This methyl group conjugation not only affects the bioconversion pathways of many drugs but also affects the properties of endogenous neurotransmitters and hormones (4). Moreover, methylation is fundamental to regulating gene expression. Unlike DNA methylation which has been linked to gene silencing, RNA and protein methylation show differential patterns of activating and repressing gene transcription. Proteins can be methylated at different amino acids, primarily lysine and arginine residues (5, 6). Gene expression can be governed by lysine methylation on two levels: methylation of histones and non-histone proteins such as transcription factors and chromatin modifiers (7).

## 2 Structural features of SETD5

**Figure 1:**
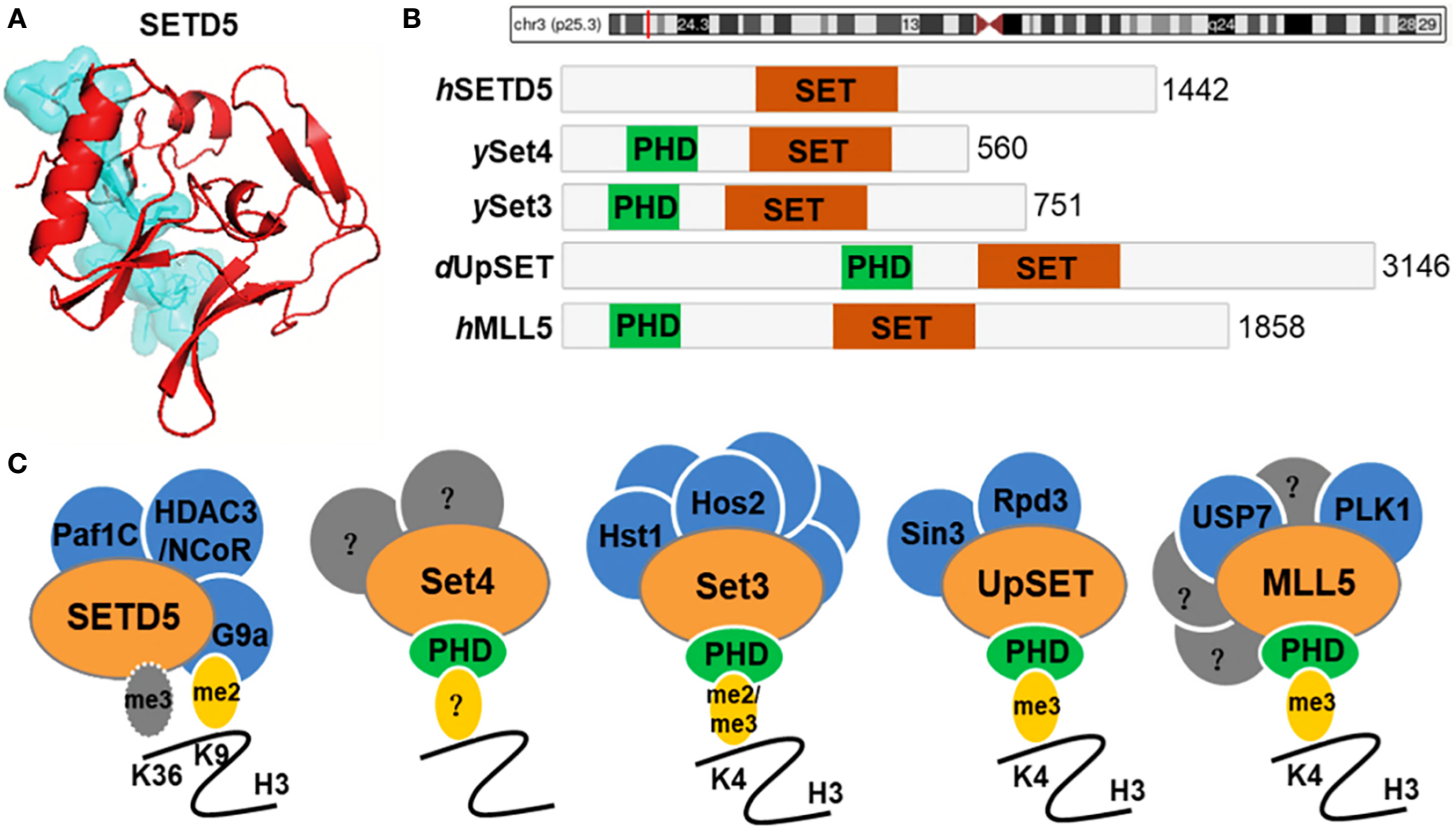
SETD5 domain composition and homologue architecture. **(A)** Crystal structure of human SETD5 protein. **(B)** Schematic indicating the protein domain organization of human (*h*) SETD5 and MLL5, yeast (*y*) Set3 and Set4, and *Drosophila* (*d*) UpSET. SET domains are shown in red and PHD fingers are shown in green. The total number of amino acids is indicated for each protein. **(C)** SETD5 contains the SET domain and is annotated as a candidate protein of lysine methyltransferase, which methylates H3K36 residue. However, there is evidence that SETD5 lacks the methyltransferase activity but scaffolds the G9a/HDAC3 co-repressor complex, which couples methylation of H3K9 with deacetylation of this residue. Members of the Set3-Set4 SET domain subfamily are shown with known interacting partners and methyl-lysine binding activity of their PHD fingers. Known binding partners are shown in blue. Set4 is predicted to interact with other factors (shown in gray) that remain to be identified. MLL5 has known interactors, a subset of which are shown in blue, and other yet-to-be-determined factors are indicated in gray.

The human *SETD5* gene (OMIM 615743), also known as MRD23, SETD5A, 2900045N06Rik or mKIAA1757, is located on the chromosome 3p25.3 and encodes the SETD5 protein composed of 1442 amino acids (8). The *SETD5* gene consists of 31 exons and is ubiquitously expressed in human tissues such as the brain, thyroid, skin, ovary, lung and endometrium (9, 10). SETD5 contains a SET (Su(var)3-9, enhancer-of-zeste, trithorax) domain and is thus annotated as a candidate protein of lysine methyltransferase, which methylates H3K36 up to the tri-methyl form (H3K36me3) (9, 11, 12) (**Figure 1**). It belongs to SET-domain lysine methyltransferase superfamily which functions to methylate certain histone lysine residues, resulting in regulating the expression of genes. However, there is evidence that SETD5 lacks the methyltransferase activity but scaffolds a co-repressor complex, including HDAC3, NCoR, G9a, and PAF1, which couples selective deacetylation of H3K9ac with methylation of this residue (13–15). The yeast SET3 and SET4, *Drosophila* UpSET, and human MLL5 are homologous to SETD5 over their SET domains and, except for SETD5, contain a PHD finger (**Figure 1**). The PHD finger of MLL5 binds the H3K4me3 mark (16, 17), and *Drosophila* UpSET also recognizes H3K4me3 (16).

## 3 Biochemical features of SETD5

The main role of SETD5 is gene activation by trimethylating H3K36 residue (**Table 1**). In this reaction, SETD5 utilizes the cofactor SAM as a methyl group donor, which binds to the substrate-binding site of the SET domain (9). In contrast, SETD5 can induce the methylation of H3K9 independently of its SET domain. This is achieved by binding to G9a histone methyltransferase and HDAC3 histone deacetylase complex, thus forming a SETD5-G9a-HDAC3 co-repressor complex (13). SETD5 also deacetylates H3K9ac; when partnered with HDAC3/NCoR1, SETD5 is converted from a relatively promiscuous enzyme into a selective one (13). This implies a model in which the SETD5-G9a-HDAC3-NCoR1 co-repressor complex couples selective methylation of H3K9 with deacetylation of this residue at target genes. Furthermore, SETD5 recruits the HDAC3 complex to the rDNA promoter, resulting in the removal of H4K16ac and its reader protein TIP5, a repressor of rDNA expression (18) (**Table 1**). Another finding by Villain et al. was the connection of SETD5 with BRD2, a bromodomain protein that recruits transcription regulators onto the chromatin (19). In more detail, both SETD5 and BRD2 bind to upstream promoter regions of the *Sema3A* locus and BRD2 is necessary for regulating *Sema3A* expression by SETD5.

**Table 1:** Summary of the identified SETD5 substrates.

Complex	Substrate	Methylation sites	Acetylation sites	Effect of the modification	Reference
Unknown	Histone H3	K36	/	Preservation of global transcriptional fidelity during brain development and neuronal wiring	(9)
G9a, HDAC3, NCoR1	Histone H3	K9	/	Promoting H3K9 methylation *via* interacting with G9a/HDAC3/NcoR1 complex and enhancing PDAC resistance to MEKi	(13)
HDAC3, NCoR, PAF1	Histone H3	/	K27	Promoting H3K27 deacetylation *via* recruiting HDAC3/NCoR co-repressor and suppressing adipogenesis	(14)
HDAC3	Histone H4	/	K16	Elevating rDNA expression *via* an HDAC3-mediated H4K16 deacetylation and promoting neural cell proliferation	(18)

K, lysine; MEKi, MEK1/2 inhibition; PDAC, pancreatic ductal adenocarcinoma.

**Figure 2:**
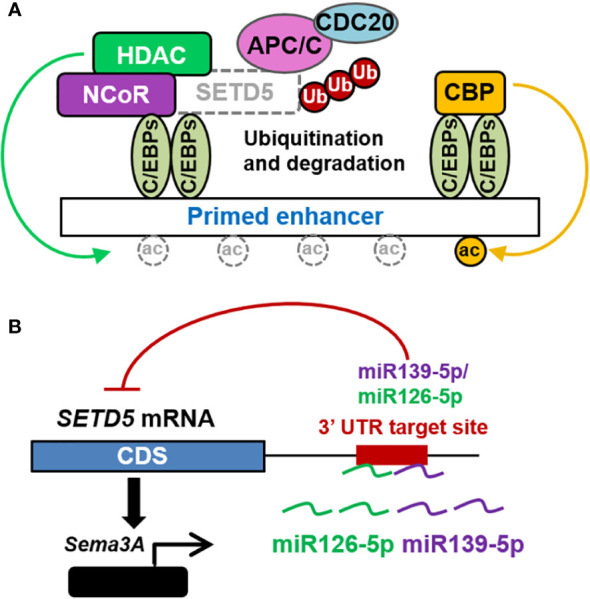
Regulatory mechanisms of SETD5 expression and activity. **(A)** SETD5 in NCoR-HDAC complex on primed enhancers is ubiquitinated and degraded by APC/C. The degradation of SETD5 from NCoR-HDAC3 co-repressor complex allows H3K27 acetylation and transits enhancers from primed to active state. **(B)** miR-139-5p or miR-126-5p binds to the *SETD5* gene 3’ untranslated region (UTR) to repress the expression of *SETD5* leading to low expression levels of *Sema3A*.

Several mechanisms have been proposed to regulate SETD5 expression and activity. The nuclear localization signal (NLS) motif in SETD5 protein can control its nuclear levels. Another interaction that can handle the nuclear levels of SETD5 is its degradation by the proteasome *via* the APC/C E3 ubiquitin ligase (14) (**Figure 2A**). Furthermore, SETD5 expression is inhibited by miR-139-5p, which may be sponged by circRNA PTPRM (circPTPRM) (20). SETD5 is also downregulated by miR-126-5p, which represses the expression of neuron-related genes in neurons (19, 21); however, the importance of this mechanism remains to be explored (**Figure 2B**).

## 4 Physiologic functions of SETD5

### 4.1 SETD5, directly and indirectly, affects cellular functions

**Figure 3:**
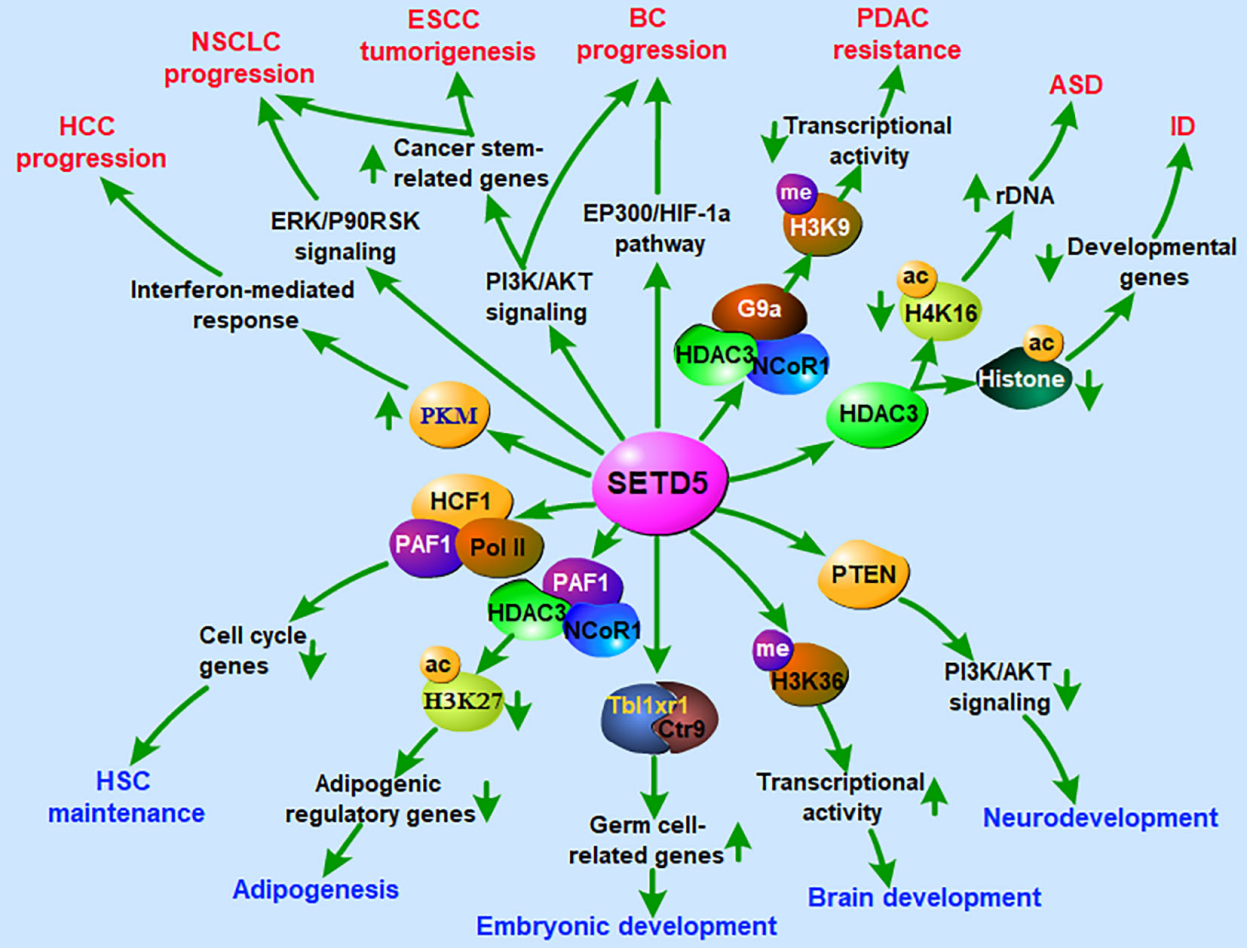
Roles of SETD5 in regulation of nervous system development, embryonic development and tumorigenesis. ASD, autism spectrum disorder; BC, breast cancer; ESCC, esophageal squamous cell carcinoma; HCC, hepatocellular carcinoma; HSC, hematopoietic stem cell; ID, intellectual disability; NSCLC, non-small cell lung cancer; PDAC, pancreatic ductal adenocarcinoma.

The cellular functions of SETD5 are primarily related to the trimethylation of H3K36, an active mark. Thus, SETD5 generates an “open”, more loose and accessible chromatin to transcription factors from a “closed” and inaccessible chromatin (9). SETD5 can also indirectly cause the deposition of repressive marks on histone tails by cross-talk with repressive methyltransferase. One indirect pathway of gene silencing is the interaction of SETD5 with the histone methyltransferase G9a, which dimethylated H3K9, establishing a repressive mark (13). Furthermore, the interaction of SETD5 with histone deacetylase HDAC3 causes the deposition of other repressive marks on histone tails (14). Therefore, SETD5 has the potential to interact indirectly with more pathways and repress a wider variety of genes. The change in chromatin architecture caused by SETD5, especially in gene enhancers or promoters, leads to the silencing of a vast array of genes. In these ways, SETD5 participates in several cellular functions, including regulation of the cell cycle and cell proliferation (22, 23), regulation of RNA elongation and splicing (9), control of brain and nervous system development (9, 19, 24–27), maintenance of tissue homeostasis (28–31), and embryonic development (22, 32, 33) (**Figure 3**). Recently, SETD5 has been extensively associated with tumorigenesis (10, 13, 23, 34–39).

### 4.2 SETD5 coordinates the nervous system development

SETD5 has been demonstrated to participate in the early development of the nervous system. At different developmental stages, SETD5 exhibits a high expression level in the cerebral cortex (40, 41). The *de novo* mutation of the *SETD5* gene has been identified as a genetic cause of neurodevelopmental disorders, such as intellectual disability (ID), autism spectrum disorder (ASD), and KBG syndrome (27, 42–46) (**Figure 3**). Loss-of-function mutations in *SETD5* lead to intellectual impairments often associated with speech, language, and developmental motor delays (8, 40, 47–49). Psychiatric manifestations of ASD-like behavior and obsessive-compulsive disorder (OCD) with hand flapping and ritualized movements have also been reported in *SETD5* patients (40, 47, 50–52). Furthermore, the dysregulation of the axis SETD5-H3K36me3 is responsible for the alteration of neural progenitor proliferation and the synapse impairment that leads to neurological symptoms (9). It has been recently proposed that ASD may develop from altered mechanisms affecting neural progenitors (40), suggesting that SETD5 may act as a key regulator in ASD development.

### 4.3 SETD5 regulates the embryonic development

Another major cellular effect of SETD5 is the regulation of embryonic development. During early embryogenesis, SETD5 is required for maintaining the expression of germ cell-related genes and SETD5-associated protein complexes containing Tbl1xr1 and Ctr9, which in turn are involved in regulating the germ cell-related genes in murine ESCs (33) (**Figure 3**). Deletion of *SETD5* results in embryonic lethality at embryonic days 10.5 and 11.5 (22). In more detail, *SETD5*-deficient mouse embryos exhibit severe defects in neural tube formation, somitogenesis and cardiac development and have aberrant vasculogenesis in embryos, yolk sacs and placentas. Furthermore, the haploinsufficiency of *SETD5* leads to disrupted developmental gene expression and cognition (41, 53). These data suggest a potential role of SETD5 in early embryonic development.

### 4.4 Connection of SETD5 with tumorigenesis

Knowledge about the function of SETD5 in tumors is sparse, and most of the information available is about its role in neurodevelopmental diseases (**Figure 3**). *SETD5* is located on chromosome 3p25.3 in a region linked to various diseases and amplified in primary tumors (8, 54, 55). Genomic alterations of *SETD5* occur in multiple cancer types, implicating its cancer-promoting role (56, 57). In most cases, the upregulation of SETD5 is detected in pancreatic cancer, breast cancer, esophageal squamous cell carcinoma (ESCC), and non-small cell lung cancer (NSCLC) (13, 23, 56, 57). The high levels of the *SETD5* gene are related to poor prognosis in patients with lung, bladder, and prostate cancer (35, 38, 56, 57). By contrast, the suppression of SETD5 expression leads to reduced cell growth and migration in pancreatic cancer, prostate cancer, and hepatocellular carcinoma (HCC) (13, 34, 58), as well as enhanced resistance to chemotherapeutic drugs (13). In terms of the mechanism, SETD5 is proposed to act as a tumor driver by inhibiting tumor suppressor gene transcription through H3K9 methylation *via* interacting with G9a/HDAC3 complex (13). Another mechanism of SETD5 involvement in cancer is the regulation of cell cycle-related genes through activating the PI3K/AKT signaling pathway (10, 23, 56) (**Figure 3**).

In addition, mutations or amplification in the SET-domain proteins has been previously reported in various cancers. According to data in the PECAN database (https://pecan.stjude.cloud/home), high-grade gliomas and acute lymphoblastic leukemias present *SETD5* mutations. *SETD5* gene mutations are also associated with prostate cancer, colorectal cancer, and neuroblastoma (36, 59–61). Furthermore, SETD5 is identified with a rate of high-level amplification at around 10% in bladder cancer (38). Either mutation or amplification is demonstrated to promote the proliferation of cancer cells (38, 60).

Recent reports shed more light on how altered SETD5 activity promotes tumorigenesis and progression. These studies investigated the role of SETD5 in breast cancer, ESCC, and NSCLC (10, 23, 37). In more detail, SETD5 acts as a factor to reprogram stemness-related gene expression patterns. The deletion of *SETD5* induces the inactivation of the PI3K/AKT pathway (10, 23). This leads to the repression of stemness-related genes like SOX2, CD44, and OCT4, which reduce stem cell-like properties and malignant transformation.

## 5 Outlook

Despite the recent achievements in the structural and biochemical analyses of SETD5 protein, not much information is available on its cellular functions. Nevertheless, the evidence that the methylation of H3K36 plays an important role in regulating enhancer activity and SETD5 is amplified in many cancers suggests that SETD5 must play a pivotal role in many different cellular processes. Epigenetic-based therapies are emerging as effective and valuable approaches in cancer, and targeting SETD5 may present a practical approach. Further research on the discovery and use of SETD5 inhibitors to combat cancer subtypes could help maximize the effects of current therapeutic regimens. First, a deeper understanding of the enzyme’s intracellular effects and affected genes is needed since there is evidence that SETD5 may also act as a tumor driver in some stages of cancer development. The cross-talk of SETD5 with other epigenetic enzymes also needs further exploration to minimize off-target side effects from its therapeutic targeting.

## Author contributions

ML: writing original draft and editing. YH: writing-original draft. ZZ: investigation. BZ: writing original draft. TH: writing original draft. AS: review and editing. GS: writing-review and editing and supervision and funding acquisition. QL: editing and supervision and funding acquisition. All authors contributed to the article and approved the submitted version.
